# Metabolic Health and Fitness Do Not Differ Substantially Between Overweight Adults With and Without α‐Actinin‐3 Deficiency

**DOI:** 10.1096/fj.202600123RR

**Published:** 2026-04-25

**Authors:** Tomas Venckunas, Thomas Chaillou, Hans Degens, Andrejus Subocius, Petras Minderis, Arvydas Stasiulis, Viktorija Maconyte, Dalia Mickeviciene, Mantas Mickevicius, Audrius Snieckus, Aurika Vanckaviciene, Leonardo Cesanelli, Hakan Westerblad, Sigitas Kamandulis

**Affiliations:** ^1^ Lithuanian Sports University Kaunas Lithuania; ^2^ Institute of Metabolic and Cardiovascular Diseases INSERM/Université de Toulouse, Team MetaDiab Toulouse France; ^3^ Department of Life Science Manchester Metropolitan University Manchester UK; ^4^ Department of Nursing, Medical Academy Lithuanian University of Health Sciences Kaunas Lithuania; ^5^ Department of Physiology and Pharmacology Karolinska Institutet Stockholm Sweden

**Keywords:** body composition, cardiopulmonary fitness, FatMax, glucose tolerance, insulin sensitivity, lipid metabolism, skeletal muscle, VO_2_max

## Abstract

The common *ACTN3* R577X polymorphism leads to α‐actinin‐3 deficiency in ~20% of the human population and may be detrimental to their muscle power. However, the impact of *ACTN3*R577X on metabolic health and exercise capacity in the general population remains unclear. The objective of the current study was to compare metabolic health markers, musculoskeletal traits, and cardiorespiratory capacity between untrained overweight α‐actinin‐3‐deficient (XX; 20 men and 19 women) and α‐actinin‐3 expressing (RR; 20 men and 21 women) individuals. The participants were aged 43 ± 7 years and had a BMI of 28.6 ± 3.2 kg·m^−2^. Various metabolic health and exercise capacity aspects encompassing segmental body composition, bone density, systemic low‐grade inflammation, blood lipid profile, whole‐body glucose tolerance and insulin sensitivity, resting and exercise metabolism, and exercise capacity were evaluated. While XX groups had lower fat‐free mass than RR groups, other anthropometrical and body composition features, including bone mineral content, did not differ between the genotype groups in either women or men. We found no significant differences between XX and RR individuals for blood lipid profile, markers of systemic inflammation, glucose tolerance, resting metabolism, and leg strength. Moreover, no clear genotype‐related differences were observed in markers of insulin resistance and sensitivity, although XX women exhibited a slightly smaller increase in insulin concentration than RR women during an oral glucose tolerance test. An incremental cardiopulmonary cycling test revealed no differences in metabolic and heart rate responses, maximal fat oxidation, or exercise capacity. In conclusion, we observed no associations between α‐actinin‐3 deficiency and metabolic health, body composition, muscle function, or cardiorespiratory capacity in untrained overweight men and women.

## Graphical abstract

Untrained overweight α‐actinin‐3‐deficient (XX; 20 men, 19 women) and α‐actinin‐3‐expressing (RR; 20 men, 21 women) participants were included in the study. No major differences were observed between the XX and RR groups for metabolic health markers, anthropometrical and body composition features, cardiopulmonary capacity, and muscle strength. Notably, XX women showed a smaller insulin increase during the oral glucose tolerance test, and XX individuals exhibited lower fat‐free mass compared with RR counterparts.

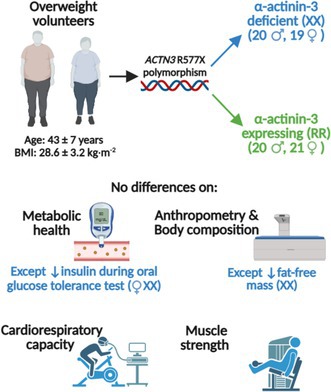

## 1 Introduction

Skeletal muscle is a primary site for energy use and storage, accounting for up to ~50% of body mass [1] and 20%–30% of the basal metabolic rate [2]. Weight gain/obesity is associated with serious health complications, including an increased risk of developing cardiovascular disease, type 2 diabetes mellitus, cancer, and metabolic syndrome [3]. Although skeletal muscle is a major contributor to whole‐body metabolism and energy expenditure, little is known about how polymorphisms of skeletal muscle genes influence body composition and the risk of metabolic diseases.

The skeletal muscle α‐actinins (coded by *ACTN2* and *ACTN3*) are actin‐cross‐linking proteins [4] with high sequence similarity [5, 6]. Alpha‐actinin‐3 is specifically expressed in type 2 (fast, glycolytic) fibers [7]. The homozygosity for a common loss‐of‐function polymorphism (XX) in *ACTN3* [R577X, rs1815739] results in a complete absence of α‐actinin‐3 in at least 1.5 billion people worldwide [8], while the expected impact on skeletal muscle susceptibility to damage, possibly affecting whole body metabolism, is likely to a large extent compensated by elevated expression of α‐actinin‐2 [9, 10, 11]. In line with this, loss of α‐actinin‐3 is not overtly associated with any disease, but has been linked to lower muscle mass, force, and power [12, 13, 14, 15]. In contrast, loss of α‐actinin‐3 may confer advantages for endurance phenotypes [16]. However, the association between *ACTN3* genotype and athletic phenotypes [12, 14, 17, 18, 19, 20, 21, 22, 23, 24, 25] is not consistently observed, and the role of the 577X allele therefore remains incompletely understood. In particular, the impact of the *ACTN3* genotype on metabolic health and physical fitness in non‐athletic populations remains unclear.

Besides a potential impact of the *ACTN3* genotype on muscle structure and function, it has been shown that α‐actinin‐3‐deficient humans and mice maintain a higher core body temperature during cold exposure due to increased muscle tone rather than overt muscle shivering [26]. This suggests that the loss of α‐actinin‐3 may be beneficial for humans in colder climates, helping them maintain body temperature. On the other hand, type 2 diabetes was more frequent among obese *ACTN3* 577XX individuals than among R‐allele carriers [11], and body fat percentage was higher among XX than RR recreational marathon runners [27]. This suggests that while α‐actinin‐3 deficiency may help to maintain body temperature, it may be detrimental for metabolic health and cardiorespiratory capacity in environments characterized by easy access to calorie‐dense foods and minimal cold exposure.

The aim of the current study was therefore to compare metabolic health markers, musculoskeletal traits, and cardiorespiratory capacity between overweight untrained XX and RR individuals. We hypothesize that XX individuals will show impaired fitness and metabolic health compared to RR individuals. Specifically, we anticipated that at least some aspects of body composition, resting fat oxidation, glucose handling, muscle function, and cardiorespiratory capacity would be compromised in XX compared with RR individuals.

## 2 Methods

### 2.1 Participants and Organization of the Study

Three hundred healthy, untrained 30‐ to 60‐year‐old individuals with a body mass index (BMI) of 25–35 kg·m^−2^ agreed to participate in the study and provided saliva samples for genotyping along with their written consent for participation. Following genotyping, 21 men and 21 women with the *ACTN3* XX genotype, along with an equal number of age‐matched RR genotype men and women, were enrolled. Four of these participants did not complete all the tests and measurements, resulting in a final sample size of 19–21 individuals per group. Participant characteristics are provided in Table 1. Exclusion criteria were an established cardiovascular or metabolic disease, cancer, or any other disease or condition precluding participation in any of the tests and measurements of the study. Moreover, subjects were excluded if they were on a weight reduction program, were exercising more than 3 h per week, or had experienced a body mass loss of more than 5 kg over the last year.

**TABLE 1:** Anthropometric and muscle characteristics across genotype groups.

	Men		Women		*P* value		
	RR (*n* = 20)	XX (*n* = 20)	RR (*n* = 21)	XX (*n* = 19)	Genotype effect	Sex effect	Interaction
Age, yrs	41.5 ± 7.2	43.7 ± 6.8	41.9 ± 6.7	44.5 ± 8.6	0.143	0.720	0.887
Height, m	1.84 ± 0.07	1.82 ± 0.06	1.68 ± 0.07	1.68 ± 0.07	0.360	< 0.001	0.642
Body mass, kg	97.8 ± 16.5	96.0 ± 11.4	84.1 ± 12.6	76.6 ± 8.9	0.105	< 0.001	0.314
Body mass index, kg·m^−2^	28.8 ± 4.1	28.7 ± 2.5	29.5 ± 3.2	27.2 ± 2.2	0.103	0.440	0.077
Waist circumference, cm	103 ± 11	104 ± 6	97 ± 5	95 ± 9	0.680	< 0.001	0.355
Hip circumference, cm	109 ± 7	109 ± 6	114 ± 7	109 ± 5	0.135	0.132	0.174
Waist/Hip	0.94 ± 0.05	0.95 ± 0.05	0.86 ± 0.04	0.86 ± 0.06	0.354	< 0.001	0.840
Knee extension peak torque, Nm	257 ± 41	248 ± 44	176 ± 33	157 ± 32	0.114	< 0.001	0.569
Knee flexion peak torque, Nm	125 ± 26	114 ± 26	78 ± 13	71 ± 14	0.061	< 0.001	0.630
m. vastus lateralis thickness, mm	27.3 ± 3.4	27.6 ± 4.0	25.3 ± 3.8	24.7 ± 4.8	0.888	0.008	0.622

There were three visits to the lab for those selected for the study. The first visit took place after an overnight fast of 10–12 h to measure resting metabolism, lipid profile, and C‐reactive protein (CRP), and oral glucose tolerance. For the second visit to the laboratory, participants arrived at least 2 h after their last meal to undergo anthropometrical measurements, muscle strength tests, and cardiopulmonary exercise tests. A dual‐energy x‐ray absorptiometry (DXA) scan was performed on a separate day without vigorous exercise in the 12 h before the scan, and at least 4 h after the last meal and drink. The study was approved by the Regional Biomedical Research Ethics Committee (permit no. BE‐2‐9).

### 2.2 Genotyping

DNA was obtained from the oral epithelium of saliva collected into sterile plastic tubes, with participants having not eaten for at least 1 h. DNA extraction was conducted using the PureLink Genomic DNA Mini Kit (Thermo Fisher Scientific, USA& Canada) according to the manufacturer's instructions. Before genotyping, DNA concentration and quality were assessed spectrophotometrically.

The determination of rs1815739 polymorphism (substitution C/T, or R577X) was performed on a “StepOnePlus” real‐time PCR quantification system (Applied Biosystems, Thermo Fisher Scientific, Singapore) using predesigned TaqMan Genotyping assays containing fluorescently labeled (FAM and VIC) minor groove binder (MGB) probes and primers (Thermo Fisher Scientific, Pleasanton, CA, USA) according to the manufacturer's recommendations. Genotyping results were obtained using a genotyping program on the StepOne software. Samples were genotyped in duplicate, and each genotyping run included positive controls (samples of known genotype) and negative controls (nuclease‐free water). All duplicates matched.

### 2.3 Anthropometrics

Quantification of whole‐body bone mineral density and content, as well as estimation of body composition, was performed using DXA (Hologic Horizon A, Marlborough, MA, USA) with data processing by Apex v. 5.6.0.5 software. Height, body mass, waist, and hip circumferences were measured after the oral glucose tolerance test, i.e., following at least 12 h of fasting and after participants had used the toilet. Height was measured using a stationary stadiometer, circumferences taken with a flexible tape, and body mass was measured using electronic scales (TBF–300 Tanita, Tokyo, Japan).

### 2.4 Thickness of m. Vastus Lateralis

As in our previous study [28], with the participants supine on a massage table, transverse images of the vastus lateralis and intermedius of the dominant leg were obtained using B‐mode ultrasonography with a 10–15 MHz linear transducer (Echoblaster 128, UAB; Telemed, Vilnius, Lithuania), applying minimal probe pressure. The probe placement was standardized at the midpoint between the greater trochanter and the lateral femoral epicondyle. The muscle thickness was assessed as the distance between the interfaces of adipose tissue–muscle and muscle–bone at the middle of the image. Three transverse images were acquired, and the average was calculated using ImageJ software (Wayne Rasband, NIH, Bethesda, MD, USA).

### 2.5 Resting Metabolism

Resting O_2_ uptake and CO_2_ production were measured as 5‐s averages using a metabolic cart (Omnical RMR/BMR, Maastricht Instruments, Maastricht, the Netherlands). During the measurements, room air temperature was 22.4°C ± 1.2°C and relative humidity 42% ± 6%. The cart was pre‐warmed and calibrated according to the manufacturer's instructions before each measurement and validated once a month via methanol burning. Measurements were carried out by using a transparent hood with the participant comfortably lying in a supine position on a padded couch in a thermoneutral laboratory for at least 30 min. Participants were instructed to remain calm without falling asleep. To eliminate all possible external disturbances that could trigger exacerbated responses of the sympathetic nervous system, the room was dimly lit, with no extraneous people or sounds. In addition, participants were asked to turn off their phones and put them away, and to wear clothing in which they felt thermally comfortable. The first 5 min of the recordings were discarded, and the remaining data were inspected for outliers (removed if detected) and then averaged. Data falling within 10% of the coefficient of variation were used for subsequent analysis. Carbohydrate (CHO) and fat oxidation rates were calculated using classic equations [29]. Total 24‐h energy expenditure was calculated using the energy equivalents of 4.2 kcal for 1 g of CHO and 9.1 kcal for 1 g of fat. Estimated resting energy expenditure was calculated using Mifflin's equation [30].

### 2.6 Blood Markers

Between resting metabolism measurement and the oral glucose tolerance test, capillary blood was sampled for analysis of triglycerides, total cholesterol, and HDL‐cholesterol using the soft reagents provided by Spotchem EZ SP‐4430 (Arkray, Japan) and measurement of CRP using the i‐Chroma II analyzer with the producer's provided reagents (Boditech, South Korea). Plasma atherogenic index was calculated as log_10_[triglycerides/HDL‐cholesterol].

### 2.7 Oral Glucose Tolerance Test

An oral glucose tolerance test was performed by measuring venous blood glucose, insulin, and lactate response to the ingestion of glucose. A catheter was inserted into the antecubital vein of the non‐dominant arm, and a baseline blood sample was drawn into a 3.5‐mL vacutainer tube containing silica clot‐activator and polymer gel for serum separation (Becton Dickinson, Franklin Lakes, NJ, USA).

Then participants ingested 75 g of glucose dissolved in 230 mL warm tap water, and 20 mL of concentrated lemon juice (Lemondor, Polenghi FOOD srl, San Rocco al Porto, Italy) was added. Venous blood sampling was then done every 15 min until 120 min. The glucose concentration was measured in triplicate from the whole blood immediately at each sampling point using glucose dehydrogenase strips and compatible analyzers (Contour Plus One, Ascensia, Switzerland), and lactate concentration was measured in duplicates using the Lactate Pro 2 analyzer with compatible lactate oxidase strips (Arkray, Kyoto, Japan). Measurements per time point were averaged.

After allowing blood to clot for 30–60 min at room temperature, tubes were centrifuged at 2,000 *g* for 15 min at 4°C, and the serum was aliquoted into 1.5‐mL tubes (FL Medical, Italy) and transferred to −80°C pending insulin analysis. Insulin concentrations were measured using enzyme‐linked immunosorbent assay kits (Cat. no. KAP1251, DIA source ImmunoAssays S.A., Nivelles, Belgium) and a Spark multimode microplate reader (Tecan, Grödig, Austria) in duplicates, and the values were averaged. The coefficient of variation for glucose, lactate, and insulin concentrations was 1.9%, 2.5%, and 6.3%, respectively. The incremental area under the curve (iAUC) for glucose and insulin concentrations was calculated using the trapezoidal method. iAUC was computed as the area above baseline glucose and insulin concentrations between 0 and 120 min after glucose intake. The Homeostatic Model Assessment of Insulin Resistance (HOMA_IR_) was calculated as fasting glucose (mmol·L^−1^) × fasting insulin (μIU·mL^−1^)/22.5 [31]. Matsuda's whole‐body insulin sensitivity index was calculated from glucose and insulin during the oral glucose tolerance test [32]. HOMA_IR_ was used to estimate fasting hepatic insulin resistance, while the Matsuda whole‐body insulin sensitivity (IS) index was calculated to capture both hepatic and peripheral insulin action.

### 2.8 Dynamometry

Concentric isokinetic peak torque of the knee extensor and flexor muscles was measured for both legs using an isokinetic dynamometer (System 4; Biodex Medical Systems, Shirley, NY, USA). Before testing, participants performed a 5‐min warm‐up by cycling on a stationary ergometer (Cardio XT6 BT, Hammer Sport AG, Neu‐Ulm, Germany) with resistance set to generate up to 60 W. The participants were instructed to select duration and intensity according to their needs to gradually increase muscle temperature without inducing fatigue. Then, participants were strapped with a double shoulder seat belt to stabilize the upper body. The distal ends of the thigh and shank were strapped to the seat and the dynamometer arm, respectively. The rotational axis of the dynamometer was aligned with the knee joint axis. Each participant performed three maximal consecutive concentric movements of knee extension and knee flexion at an angular velocity of 60°·s^−1^ with their right and then left leg. Sampling rate was 100 Hz. Peak torque values for extension and flexion were averaged across the legs for analysis.

### 2.9 Cardiopulmonary Exercise Test

The participants performed an incremental step cycling test (Corival, Lode, Groningen, the Netherlands). Pedaling cadence was kept at ~60 rpm throughout the test. First, women cycled for 4 min at 20 W and men at 30 W, then women completed a 4‐min stage at 30 W and men at 50 W, and then the workload increased with every 4‐min step by 20 W for women and by 25 W for men until volitional exhaustion, i.e., until the cadence remained lower than 50 rpm for 10 s despite continuous encouragement to reach 60 rpm. Expired air was analyzed breath‐by‐breath (Cortex MetaMax3B, Cortex Biophysik GmbH, the Netherlands). The duration of the stages (4 min) was selected to allow achievement of a steady state for the measured parameters and to permit proper estimation of work efficiency and fat oxidation.

Heart rate was recorded throughout the test using H10 transducers (Polar, Finland). Tidal volume, breathing frequency, pulmonary ventilation, oxygen uptake (VO_2_), and carbon dioxide production (VCO_2_) were recorded. Data were averaged over 60 s at rest before the test initiation and over the last 60 s of every step of the test until RER exceeded 1.00. Gross cycling efficiency was calculated as previously described by Moseley et al. [33]. Fat oxidation (in g·min^−1^) was calculated as 1.695 × VO_2 −_ 1.701× VCO_2_ (both in L·min^−1^) [34]. Maximal fat oxidation rate (the highest fat oxidation calculated at any exercise intensity, i.e., no interpolation) and corresponding cycling power were analyzed and compared between the groups.

For the last fully completed 4‐min stage (and the next initiated but not completed step, if it was the case), the 20‐s rolling average was calculated for oxygen uptake (VO_2_) to determine maximal oxygen uptake (VO_2_max), and other peak cardiopulmonary parameters (heart rate, oxygen pulse, tidal volume, breathing frequency, pulmonary ventilation, RER). Peak power output was calculated as the power of the last fully completed step plus the power equivalent fraction of the time during the next incomplete step. Capillary blood lactate concentration (Lactate Pro 2; Arkray, Kyoto, Japan) was measured before the test and at the end of each step of the test.

### 2.10 Statistical Analysis

**FIGURE 1:**
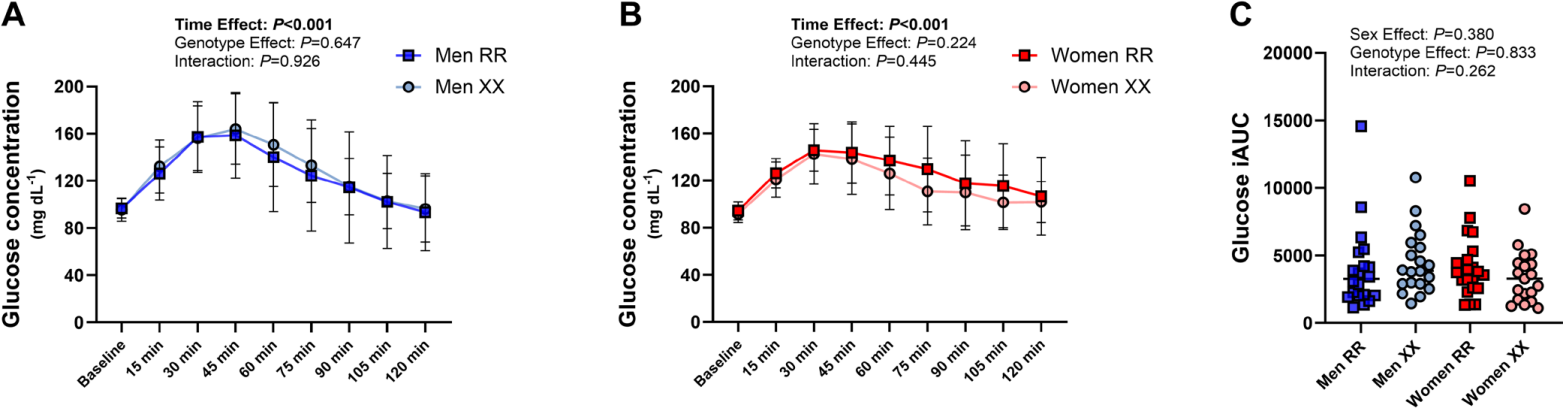
Blood glucose concentration dynamics (A—men, B—women) and incremental area under the curve (iAUC, C) during the oral glucose tolerance test (*n* = 19–21 per group).

**FIGURE 2:**
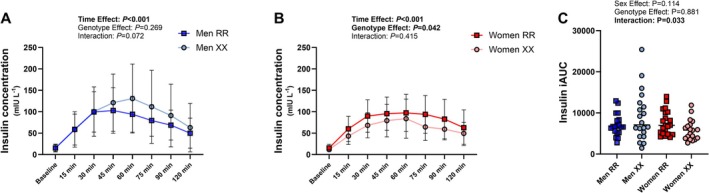
Serum insulin concentration dynamics (A—men, B—women) and incremental area under the curve (iAUC, C) during the oral glucose tolerance test (*n* = 19–21 per group).

**FIGURE 3:**
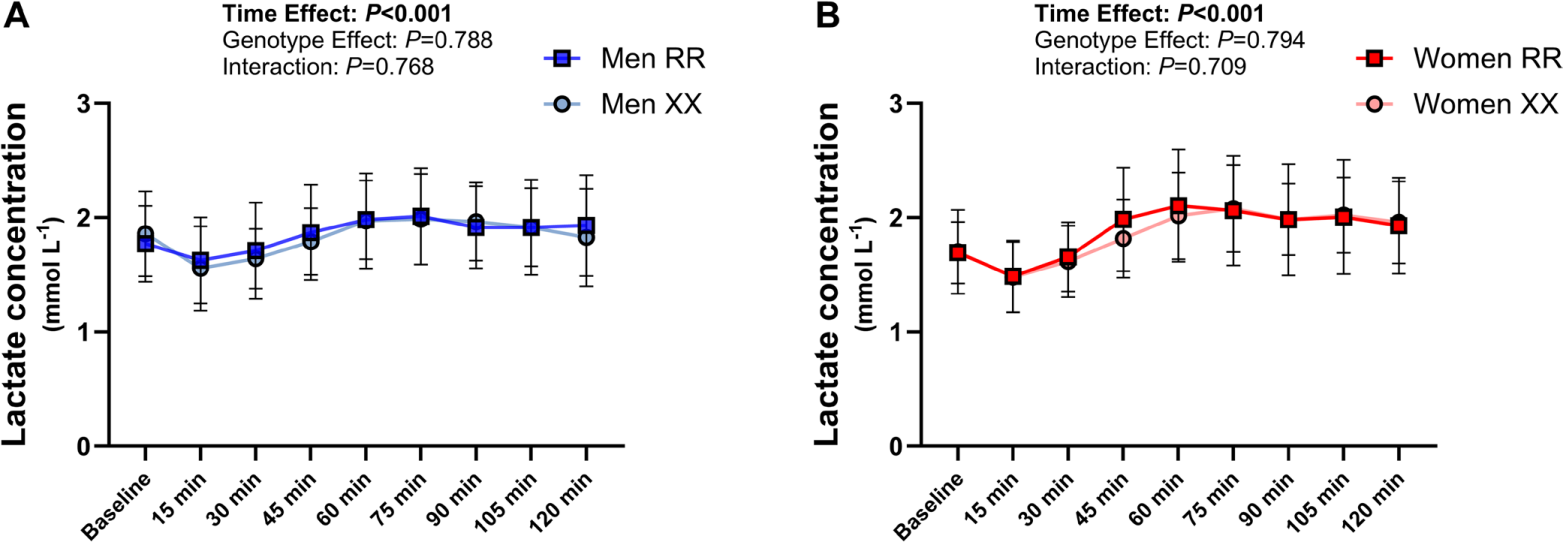
Venous blood lactate concentration dynamics (A—men, B—women) during the oral glucose tolerance test (*n* = 19–21 per group).

**FIGURE 4:**
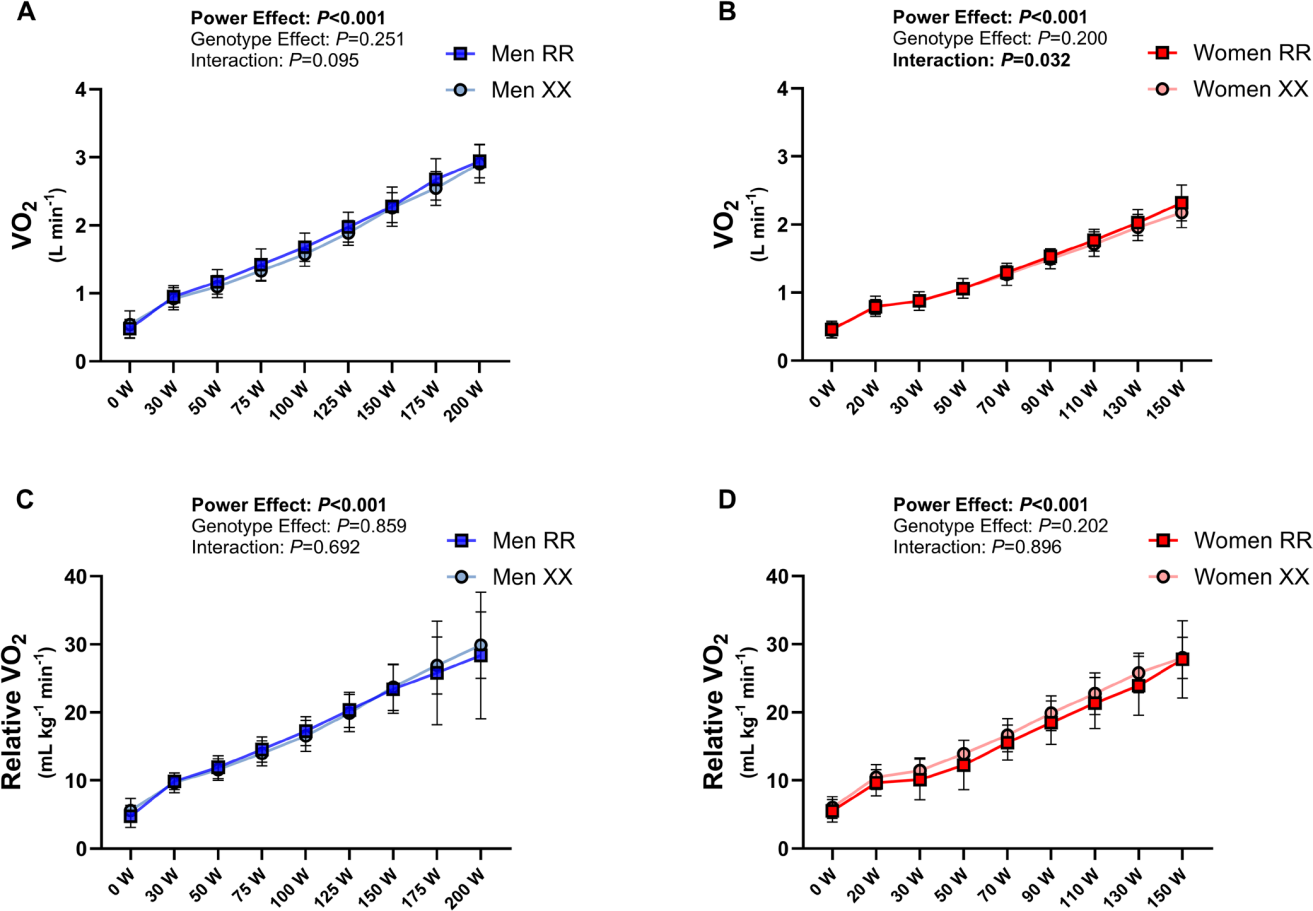
Oxygen uptake rate during submaximal incremental cycling across genotypes (*n* = 19–21 per group) in men (panels A and C) and women (panels B and D).

**FIGURE 5:**
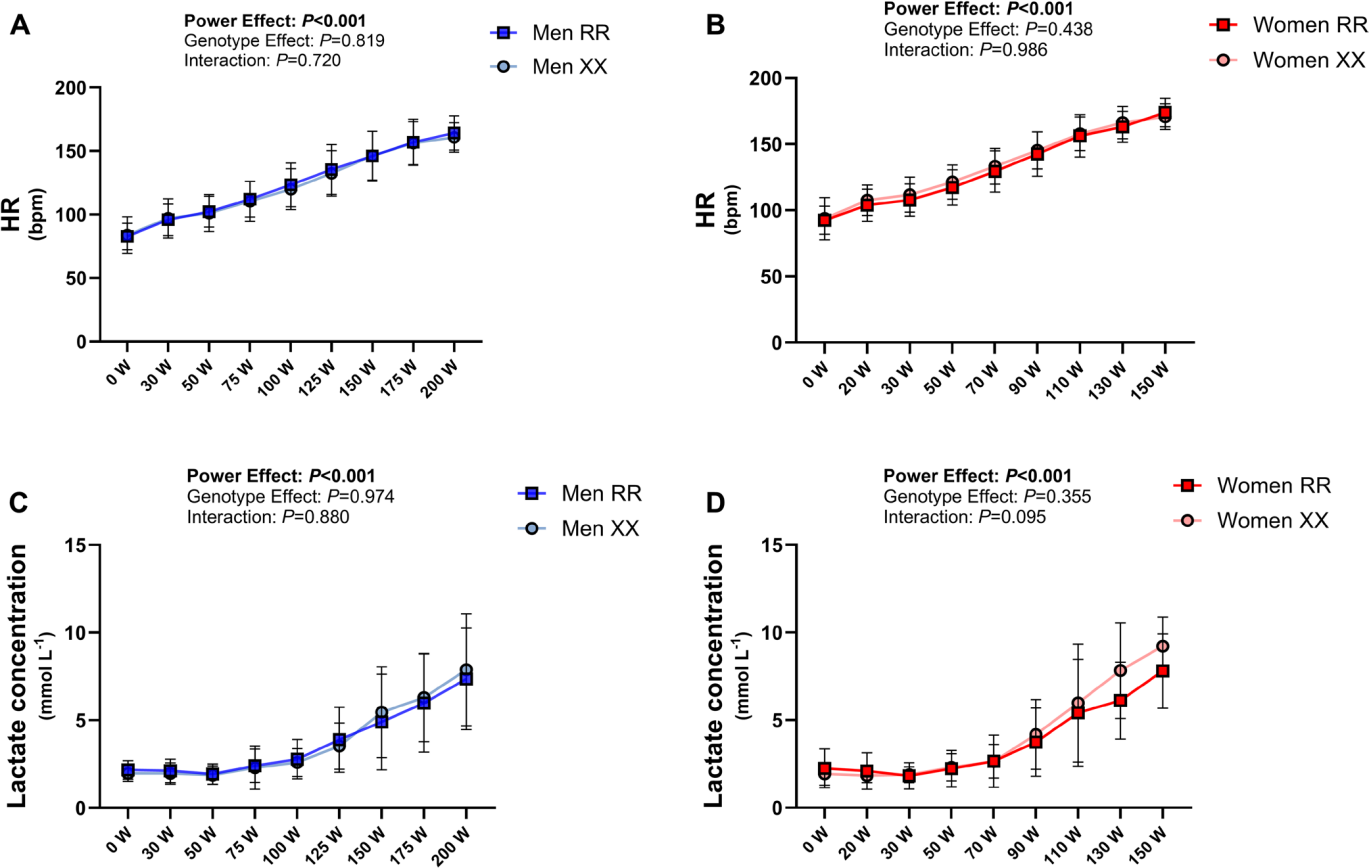
Heart rate (HR, A—men, B—women) and capillary blood lactate (C—men, D—women) responses to submaximal incremental cycling across genotypes (*n* = 19–21 per group).

Data are presented as mean ± standard deviation (SD), unless specified otherwise Figures 1C and 2C, also show individual values. All statistical analyses were performed using GraphPad Prism 10.3.1 (GraphPad Software, San Diego, USA). Data shown in the tables and in Figures 1C and 2C, were analyzed by two‐way ANOVA to assess the effects of genotype, sex, and their interaction. For analyses performed separately in men and women (Figure 1A–B, Figures 2A–B and 3–5), mixed ANOVA or mixed‐effects models (to account for missing values) were used to evaluate the effects of genotype, time, or power, and their interaction. When a significant interaction was detected, Sidak's multiple comparisons test was applied to compare XX and RR groups. Three‐way ANOVA was also performed to assess the effects of genotype, sex, and time, as well as their interactions. When applicable, Geisser–Greenhouse corrections were used to correct for violation of the sphericity assumption. Owing to the exploratory nature of the study and the broad range of outcomes assessed, no adjustment for multiple comparisons was applied to the primary statistical analyses (ANOVA or mixed‐effects models). The level of significance was set at *p* < 0.05. For the analysis of knee flexion peak torque, one woman with the RR genotype was excluded because her value was considered an outlier (> 2SD above the mean). For the same reason, one man with the RR genotype was excluded from the analysis of insulin concentration, HOMA_IR_ index, and Matsuda‐IS index. When appropriate, Cohen's d effect sizes were calculated, and effect sizes were classified as small (׀d׀ from 0.2 to 0.5), moderate (׀d׀ from 0.5 to 0.8), and large (׀d׀ above 0.8).

## 3 Results

Age, anthropometric indices, muscle force, and vastus lateralis thickness were not different between RR and XX genotypes (Table 1). Most of these parameters, except for age, body mass index, and hip circumference, were lower in women than in men.

Body composition did not differ significantly between RR and XX genotypes, except for fat‐free mass, which was larger in RR compared to XX individuals, irrespective of sex (Table 2). For fat‐free mass, the 95% confidence intervals (95% CI) were 65.1–73.7 kg in RR men, 63.2–70.1 kg in XX men, 47.9–53.6 kg in RR women, and 44.2–49.0 kg in XX women. The corresponding Cohen's d effect sizes were 0.362 in men and 0.745 in women. Visceral adipose tissue mass and bone mineral content and density did not differ between RR and XX genotypes in either men or women. Fat‐free mass, visceral adipose tissue mass, bone mineral density, and bone mineral content were higher in men than in women, while body fat percentage was higher in women (Table 2).

**TABLE 2:** Body composition, bone density, resting metabolism, blood lipids, C‐reactive protein, and indices of glucose‐insulin homeostasis across genotype groups.

	Men		Women		*P* value		
	RR (*n* = 20)	XX (*n* = 20)	RR (*n* = 21)	XX (*n* = 19)	Genotype Effect	Sex Effect	Interaction
Fat mass, kg	29.5 ± 7.5	30.2 ± 5.6	34.2 ± 7.7	30.6 ± 5.6	0.330	0.087	0.163
Fat, %	29.6 ± 3.3	31.0 ± 3.3	40.0 ± 3.7	39.5 ± 4.1	0.550	< 0.001	0.245
VAT mass, kg	0.59 ± 0.23	0.64 ± 0.18	0.50 ± 0.20	0.43 ± 0.19	0.823	< 0.001	0.187
Fat‐free mass, kg	69.4 ± 9.1	66.7 ± 7.4	50.7 ± 6.1	46.6 ± 5.0	0.035	< 0.001	0.653
Bone mineral density, g·cm^−2^	1.25 ± 0.09	1.24 ± 0.10	1.16 ± 0.07	1.12 ± 0.11	0.222	< 0.001	0.407
Bone mineral content, kg	3.15 ± 0.42	3.07 ± 0.37	2.52 ± 3.03	2.36 ± 3.10	0.151	< 0.001	0.627
Estimated resting metabolic rate (Mifflin's eq.), kcal/24 h	1926 ± 190	1882 ± 151	1530 ± 167	1431 ± 124	0.050	< 0.001	0.445
Measured resting metabolic rate, kcal/24 h	1968 ± 331	1940 ± 249	1591 ± 162	1496 ± 161	0.253	< 0.001	0.525
Fat oxidation at rest, %	66.1 ± 16.3	58.8 ± 12.0	54.4 ± 14.5	63.6 ± 20.5	0.792	0.344	0.024
CRP, mg·L^−1^	1.09 ± 1.42	0.80 ± 0.49	2.21 ± 2.39	1.51 ± 1.78	0.151	0.017	0.428
Total cholesterol, mmol·L^−1^	5.0 ± 1.2	5.0 ± 1.1	4.9 ± 0.9	5.2 ± 1.0	0.422	0.794	0.495
HDL cholesterol, mmol·L^−1^	1.25 ± 0.35	1.39 ± 0.27	1.77 ± 0.45	1.82 ± 0.63	0.353	< 0.001	0.658
TAG, mmol·L^−1^	1.27 ± 0.97	1.18 ± 0.50	0.92 ± 0.42	1.01 ± 0.39	0.974	0.067	0.497
Plasma atherogenic index	−0.06 ± 0.35	−0.10 ± 0.21	−0.30 ± 0.28	−0.26 ± 0.24	0.988	0.001	0.508
HOMA_IR_ index	3.75 ± 2.43	3.55 ± 1.76	3.66 ± 1.99	2.68 ± 1.18	0.170	0.264	0.355
Matsuda‐IS index	3.61 ± 1.79	3.50 ± 2.07	3.20 ± 1.27	4.58 ± 2.18	0.133	0.427	0.077

Abbreviations: CRP – C‐reactive protein; HDL – high‐density lipoprotein; HOMA_IR_ – Homeostatic Model Assessment of Insulin Resistance; IS – insulin sensitivity; TAG – triglycerides; VAT – visceral adipose tissue.

Measured resting metabolic rate was not different between RR and XX genotypes, while estimated resting metabolic rate was overall ~5% larger in RR compared to XX individuals, irrespective of sex. A sex × genotype interaction was found for fat oxidation at rest, but the post hoc analysis did not reveal any differences between the RR and XX genotypes in either men or women (Table 2).

Blood lipid profile and the level of CRP were similar between the genotype groups in both men and women (Table 2). Plasma atherogenic index was low in most participants, and the mean score was not different between the genotype groups (Table 2). CRP and HDL‐cholesterol concentrations were higher in women compared to men, while plasma atherogenic index was lower in women than in men (Table 2).

Glucose concentrations before and during the oral glucose tolerance test were similar between the genotype groups, and calculated incremental areas under the curve (iAUC) did not differ (Figure 1). Glucose concentration (kinetics and iAUC) was not affected by sex.

Insulin response during the oral glucose tolerance test was not significantly different between the two genotypes in men (Figure 2A), while a smaller increase in insulin concentration was observed in XX women compared to RR women (Figure 2B). Mean insulin concentration during the oral glucose tolerance test had a 95% CI of 46.6–68.3 μIU·mL^−1^ in XX women and 61.7–87.7 μIU·mL^−1^ in RR women, with a Cohen's d effect size of −0.671. A sex × genotype interaction was found for insulin iAUC, but the post hoc analysis did not reveal any differences between the RR and XX genotypes in either men or women (Figure 2C). HOMA_IR_ index and Matsuda‐IS index, two indices used to assess glucose‐insulin homeostasis, were not different between the genotype groups nor between women and men (Table 2). The lactate response during the oral glucose tolerance test was modest and did not differ significantly between the groups (Figure 3).

None of the peak variables obtained during the cardiopulmonary exercise test was different between the genotype groups in either men or women (Table 3). Oxygen consumption (Figure 4), heart rate, and capillary blood lactate responses did not differ significantly between the genotype groups (Table 3, Figure 5). Gross cycling efficiency was better in women than in men at 150 W, but there were no evident differences between the genotype groups in either sex (Table 3). As expected, other exercise‐related measures (VO_2max_, peak power, maximal tidal volume and pulmonary ventilation, maximal O_2_ pulse, and power at maximal oxidation rate) were higher in men compared to women (Table 3).

**TABLE 3:** Peak cardiopulmonary exercise test outcomes and gross cycling efficiency across genotype groups.

	Men		Women		*P* value		
	RR (*n* = 20)	XX (*n* = 20)	RR (*n* = 21)	XX (*n* = 19)	Genotype Effect	Sex Effect	Interaction
VO_2_max, L·min^−1^	3.14 ± 0.71	2.93 ± 0.60	2.18 ± 0.39	1.97 ± 0.41	0.079	< 0.001	0.953
Relative VO_2_max, mL·kg^−1^·min^−1^	32.3 ± 7.4	30.7 ± 6.2	26.3 ± 5.9	26.0 ± 5.7	0.928	0.007	0.904
Peak power, W	204 ± 39	197 ± 38	136 ± 27	130 ± 25	0.465	< 0.001	0.963
Peak power, W·kg^−1^	2.13 ± 0.51	2.06 ± 0.35	1.64 ± 0.38	1.73 ± 0.40	0.795	< 0.001	0.520
Maximal tidal volume, L	3.11 ± 0.57	3.04 ± 0.51	2.23 ± 0.37	2.01 ± 0.43	0.202	< 0.001	0.502
Maximal breathing frequency, L·min^−1^	34.5 ± 4.8	34.5 ± 7.3	33.2 ± 5.0	35.9 ± 8.2	0.366	0.961	0.361
Maximal pulmonary ventilation, L·min^−1^	106 ± 26	102 ± 26	73 ± 17	68 ± 17	0.363	< 0.001	0.957
Maximal O_2_ pulse, mL·beat^−1^	18.5 ± 4.2	17.5 ± 3.6	12.6 ± 2.1	11.6 ± 2.2	0.169	< 0.001	0.945
Maximal fat oxidation, g·min^−1^	0.33 ± 0.14	0.34 ± 0.18	0.30 ± 0.14	0.26 ± 0.09	0.684	0.099	0.560
Power at maximal fat oxidation, W	71.6 ± 39.6	68.2 ± 50.7	55.0 ± 40.7	35.3 ± 20.6	0.203	0.007	0.364
Maximal heart rate, bpm	173 ± 15	170 ± 15	167 ± 20	170 ± 11	0.921	0.569	0.492
Peak lactate, mmol·L^−1^	10.8 ± 3.0	10.0 ± 2.9	9.1 ± 2.7	9.6 ± 3.1	0.816	0.121	0.315
Gross cycling efficiency, %							
50 W	12.8 ± 1.8	13.6 ± 2.0	13.7 ± 1.0	14.0 ± 1.8	0.150	0.076	0.575
100 W^a^	17.4 ± 1.9	18.6 ± 1.9	17.6 ± 1.3	18.0 ± 1.7			
150 W	19.0 ± 2.3	19.1 ± 1.7	21.4 ± 2.1	21.8 ± 1.9	0.595	< 0.001	0.806

Abbreviation: VO_2_max, maximal oxygen consumption.

^a^ An average of 90 W and 110 W in women.

## 4 Discussion

Contrary to our hypotheses, the results of the current study disclose no signs of impaired fitness or metabolic health in untrained overweight but otherwise healthy α‐actinin‐3‐deficient men and women. Specifically, we observed no consistent difference in resting and exercise metabolism, glucose handling, lipid and inflammatory profile, body composition, bone mass, cardiorespiratory capacity, or muscle strength between XX and RR individuals. Our results imply that in overweight middle‐aged individuals, α‐actinin‐3 deficiency is not associated with an increased risk of developing metabolism‐related diseases, such as type 2 diabetes and atherosclerosis.

A positive association between 577R carrier status and muscle volume and strength has been reported in normal‐weight young men [12], but others found no such association [24]. 577R carrier status has been found to associate with muscle strength [35, 36] and thigh muscle volume [37, 38] in people over 55 years, but no such association was seen in women or older men in other studies [39, 40]. In fact, one study has even found that the 577X rather than the 577R allele was sarcopenia‐protective in elderly obese women [41]. In a large study in untrained adolescents, there was no *ACTN3* genotype association with body composition, muscle strength, and cardiorespiratory capacity, while muscle power was negatively associated with the 577X allele carrier status only in boys but not in girls [42].

While the XX genotype was associated with lower bone mass in the elderly [43, 44], the results of the present study show no such association in middle‐aged overweight individuals. These contradictory findings on associations with muscle strength and volume, cardiorespiratory capacity, and bone mass strongly suggest that the link between *ACTN3* genotype and musculoskeletal phenotypes is not robust or straightforward.

Some phenotypes associated with α‐actinin‐3 deficiency in the *Actn3* KO mice are a shift towards a slow oxidative phenotype, a lower glycogen phosphorylase activity, and a higher glycogen concentration within skeletal muscle fibers, as well as a higher GLUT‐4 expression and lower circulating glucose following a glucose tolerance test [45, 46]. Hitherto, this had not yet been sufficiently investigated in humans [47]. We did not detect any negative association of α‐actinin‐3 deficiency with carbohydrate handling efficacy or other metabolic health status markers (e.g., metabolic flexibility as reflected by maximal fat oxidation) in our middle‐aged overweight individuals. The increase in insulin concentration during the oral glucose tolerance test was slightly lower in XX than in RR women, which might be taken as a sign of a higher insulin sensitivity in the XX women. However, other measures of the insulin‐glucose interaction (HOMA_IR_ and Matsuda‐IS indices) did not show any significant difference between XX and RR women, hence indicating no physiologically important difference in insulin sensitivity between the two groups.

The absence of a clear association of *ACTN3* genotype with metabolic health markers in our study could be explained in several ways. Firstly, any potential differences in glucose handling and metabolic flexibility between the genotypes might be masked by a larger effect of adiposity. Secondly, our middle‐aged individuals were still largely healthy, and the detection of a metabolic health‐limiting effect of α‐actinin‐3 deficiency might require more severe obesity‐related problems and/or higher age. Indeed, most participants had normal fasted glucose (5.5 mmol·L^−1^ or lower in > 80% of individuals) and insulin (< 25 μIU·mL^−1^ in 90% of individuals) levels. In addition, the glucose concentration returned (within 2 mmol·L^−1^) to baseline in 90% of the participants, and neither the glucose nor insulin response was exaggerated in either genotype. The blood lactate response was also similar in the two genotypes, confirming normal glucose tolerance. Thirdly, since fat‐free mass correlates closely with total blood volume and muscle mass [48], and skeletal muscle tissue is the major disposal site of circulating glucose [49], a larger fat‐free mass in our middle‐aged RR individuals might have compensated for any impairment of glucose handling.

### 4.1 Limitations and Perspectives

The aim of the current study was to test whether α‐actinin‐3 deficiency was associated with a markedly increased risk of metabolism‐related disorders in overweight/obese individuals. However, our results did not show any signs of increased risks in α‐actinin‐3‐deficient men or women. While we included individuals with a rather broad range of BMI (slightly overweight to class I obesity), there were at best only trivial associations between BMI and metabolic health markers (data not shown). Nevertheless, there are other factors that are known to affect metabolic health (e.g., level of physical activity) that were not specified in the inclusion criteria. Therefore, we can only exclude clear associations between *ACTN3* genotype and major risk factors for overweight‐associated problems. Moreover, the small sample size of our study means that non‐significant findings do not necessarily imply the absence of meaningful differences.

The design of the study did not allow us to directly address the efficacy of nutrient energy assimilation and conservation in relation to *ACTN3* genotype or test the hypothesis that α‐actinin‐3‐deficient individuals in modern society with excess food supply render themselves more susceptible to gaining extra weight and accelerate the development of obesity. On the other hand, because we recruited untrained volunteers based only on their age and absence of overt diseases, and allowed for a wide BMI range from 25 to 35 kg·m^−2^, we would likely have arrived at RR and XX differing in their BMI if one of the genotype groups had a propensity to accrue soft tissue (muscle and/or fat) at a faster rate. However, this was not the case, and especially the accumulation of adipose tissue reserves relative to body mass was similar between the two genotypes in both men and women. Even if fat‐free mass was lower in the XX genotype, with the method used (DXA scanning), we cannot claim that whole body muscle mass differed as well, especially since our ultrasound measurement of the thickness of one of the major muscles did not disclose any genotype effect.

It is important to emphasize that individuals enrolled in our study were healthy and not taking medications with possibly negative effects on the muscles (e.g., statins). It therefore remains to be answered whether *ACTN3* genotype modifies effects on bone, muscles, metabolism, and cardiorespiratory capacity in unhealthy lifestyles such as nearly complete sedentarism and/or polypharmacy. This warrants further investigation because preliminary data suggest that obese XX individuals are more susceptible to type 2 diabetes [11], and frail (heart failure) XX patients have a survival disadvantage [50].

*In conclusion*, we found no substantial differences between overweight adults with and without α‐actinin‐3 deficiency in metabolic health, body composition, and exercise capacity.

## Author Contributions

T. Venckunas, T. Chaillou, H. Degens, H. Westerblad, and S. Kamandulis designed the study; T. Venckunas, T. Chaillou, A. Subocius, P. Minderis, A. Stasiulis, V. Maconyte, D. Mickeviciene, M. Mickevicius, A. Snieckus, A. Vanckaviciene, and L. Cesanelli collected and analyzed the data; T. Venckunas, T. Chaillou, H. Degens, and H. Westerblad wrote the paper.

## Funding

This work was supported by Lietuvos Mokslo Taryba (LMT), S‐MIP‐23‐107.

## Disclosure

The authors have nothing to report.

## Conflicts of Interest

The authors declare no conflicts of interest.

## Data Availability

The data that support the findings of this study are available on a reasonable request from the corresponding author. The data are not publicly available due to privacy.
